# Alterations in novel inflammatory biomarkers during perioperative cardiovascular surgeries involving cardiopulmonary bypass: a retrospective propensity score matching study

**DOI:** 10.3389/fcvm.2024.1433011

**Published:** 2024-09-27

**Authors:** Wei Zhou, He Wang, Chen Li, Qi-min Ma, Yan-hui Gu, Shu-yue Sheng, Shao-lin Ma, Feng Zhu

**Affiliations:** ^1^ Department of Critical Care Medicine, Shanghai East Hospital, Tongji University School of Medicine, Shanghai, China; ^2^ Health Department, Beijing Armed PAP Corps, Beijing, China

**Keywords:** cardiovascular surgery, inflammation, perioperative, pan-immune inflammatory value (PIV), neutrophil/lymphocyte ratio (NLR)

## Abstract

**Background:**

Cardiopulmonary bypass (CPB) triggers a strong inflammatory response in cardiovascular surgery patients during the perioperative period. This article mainly focuses on the perioperative application of novel inflammatory biomarkers in cardiovascular surgeries involving CPB.

**Methods:**

Patients were divided into a CPB group and a non-CPB group according to whether they underwent CPB during cardiovascular surgery. Novel inflammatory biomarkers and clinical results were recorded. The neutrophil/lymphocyte ratio (NLR), platelet/lymphocyte ratio (PLR), platelet × neutrophil/lymphocyte ratio (SII), and monocyte × platelet × neutrophil/lymphocyte ratio (PIV) were calculated. The primary outcomes were perioperative prognosis between the CPB and non-CPB groups. The secondary outcomes included perioperative alterations of novel inflammatory biomarkers in the CPB group and predictive values of novel inflammatory biomarkers for postoperative infection and acute kidney injury.

**Results:**

A total of 332 patients were initially included in the study. Before propensity score matching (PSM), there were 96 patients in the CPB group and 236 patients in the non-CPB group. After PSM, both groups included 58 patients each. Compared with the non-CPB group, the CPB group had a higher proportion of intraoperative transfusion of blood products (63.79% vs. 6.90%, *P* < 0.001), specifically for red blood cells (58.62% vs. 3.45%, *P* < 0.001) and plasma (41.38% vs. 1.72, *P* < 0.001), exhibited a higher drainage fluid volume within 24 h [380 (200–550) ml vs. 200 (24–330) ml, *P* = 0.002], and required longer durations of mechanical ventilation [14.3 (6.6–21.3) h vs. 5.75 (4.08–10.1) h, *P* < 0.001] and ICU stay [48.78 (44.92–89.38) h vs. 27.16 (21.67–46.25) h, *P* < 0.001]. After surgery, NLR [14.00 (9.93–23.08) vs. 11.55 (7.38–17.38), *P* = 0.043] was higher in the CPB group, while the PIV, PLR, and SII in the CPB group were lower than those in the non-CPB group on the first day after surgery.

**Conclusions:**

Cardiovascular surgeries involving CPB exhibit a poorer prognosis compared to non-CPB procedures. Novel inflammatory biomarkers, including PLR, PIV, and SII, may offer valuable insights into the degree of postoperative inflammation, with NLR emerging as a potentially reliable prognostic indicator.

## Introduction

Most cardiovascular surgeries need to be assisted by cardiopulmonary bypass (CPB). Currently, relevant studies have confirmed that CPB triggers a strong inflammatory response in patients during the perioperative period, thus affecting the prognosis of patients (1). With the development of surgical counting, an increasing number of surgical operations are being performed without CPB, but the postoperative inflammatory response remains strong. Inflammation is closely related to the prognosis of patients after cardiovascular surgery. Previous studies have attempted to combine multiple biomarkers to develop more accurate indicators to improve the clinical application of biomarkers, including the neutrophil/lymphocyte ratio (NLR), platelet/lymphocyte ratio (PLR), and systemic inflammation index (SII) (2). Furthermore, in recent years, the pan-immune inflammatory value (PIV) has also emerged as a popular indicator of inflammation. First proposed in 2022 as a novel prognostic biomarker for metastatic colorectal cancer (3), its application in cardiovascular surgery is rare. This article mainly focuses on the perioperative application of novel inflammatory markers in cardiovascular surgeries involving CPB and explores the perioperative changes in these biomarkers to guide their clinical application.

## Methods

### Study design

Patients undergoing open cardiovascular surgery in Shanghai East Hospital from August 2022 to June 2023 were included in this study. The clinical data of these patients were retrospectively analyzed, and the patients were divided into a CPB group and a non-CPB group according to whether CPB was used during the operation. The perioperative clinical results of the two groups were compared. This study was approved by the Ethics Committee of Biomedical Research at Shanghai East Hospital, Tongji University School of Medicine (Approval No: 2024YS-043). Given the observational nature of the study, the ethics committee waived the requirement for individual patient consent.

### Data collection

Preoperative data, including age, body mass index (BMI), height, underlying diseases, and laboratory test results (e.g., leukocytes, lymphocytes, monocytes, platelets, bilirubin, creatinine, etc.), were collected. We also gathered relevant intraoperative data of the patients, including intraoperative blood transfusion details. In addition, we recorded postoperative inflammatory biomarkers and clinical outcomes, such as acute kidney injury (AKI), postoperative infections, mechanical ventilation (MV) duration, ICU stay duration, and in-hospital mortality.

### Definitions

In our study, the following novel inflammatory biomarkers were utilized to assess the perioperative inflammatory response:

NLR: neutrophil/lymphocyte ratio, PLR: platelet/lymphocyte ratio, SII: platelet × neutrophil/lymphocyte ratio, and PIV: monocyte × platelet × neutrophil/lymphocyte ratio.

Furthermore, AKI is defined as any of the following: an increase in creatinine (SCr) by ≥0.3 mg/dl (≥26.5 μmol/L) within 48 h; an increase in SCr to ≥1.5 times the baseline level, which is known or presumed to have occurred within the prior 7 days; or urine volume <0.5 ml/kg/h for 6 h (4).

Postoperative infections include pulmonary infection, bloodstream infection, and urinary system infection.

### Statistical analysis

Propensity score matching (PSM) analysis is a method used to reduce selection bias between two groups of patients. We used a logistic regression model to calculate the propensity score for each patient and performed 1:1 matching between the two groups, with a caliper width of 0.2 standard deviations (SD). Baseline demographic data, preoperative data, intraoperative data, and postoperative data were compared between the two groups both before and after PSM. The results are presented as the mean ± SD or median [interquartile range (IQR)] for continuous variables, as appropriate, and as the total number (%) for categorical variables. Comparisons between groups were made using the *χ*^2^ test or Fisher’s exact test for categorical variables and the Student’s *t*-test or Mann–Whitney *U* test for continuous variables, as appropriate. The ability of NLR, PLR, SII, and PIV to predict postoperative clinical outcomes (mainly in postoperative AKI and infection) was analyzed by receiver operating characteristics (ROC) curves and the resulting area under the curve (AUC). All statistical analyses were performed with R 4.3.2 (R Foundation). A two-tailed *P* < 0.05 was considered statistically significant.

## Results

### Baseline demographic and clinical data of patients

**Figure 1:**
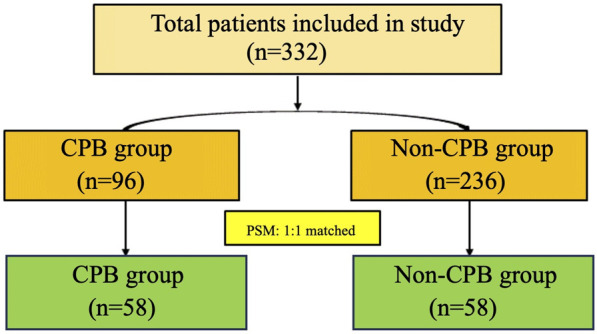
Flowchart of patient screening. PSM: propensity score matching.

The flowchart of patient screening is shown in Figure 1. Initially, 332 patients were included in the study. Before PSM, there were 96 patients in the CPB group and 236 patients in the non-CPB group. After PSM, both groups included 58 patients each.

The baseline data analysis is presented in Table 1. Before PSM, there were statistically significant differences in gender, age, New York Heart Association (NYHA) grade, and diabetes mellitus rate between the CPB and non-CPB groups. After PSM, there was no statistical difference between the two groups in terms of preoperative characteristics.

**Table 1:** Baseline demographic and clinical data between two groups.

Variable	Overall population		*P*-value	Propensity score matched population		*P*-value
	Non-CPB group (*n* = 236)	CPB group (*n* = 96)		Non-CPB group (*n* = 58)	CPB group (*n* = 58)	
Male (%)	172 (72.88)	57 (59.38)	0.023	36 (62.07)	35 (60.34)	1.000
Age (years)	67.00 [60.00, 72.25]	65.00 [54.75, 70.00]	0.012	65.00 [58.50, 70.00]	65.00 [56.50, 69.75]	0.782
BMI (kg/m^2^)	24.41 [22.45, 26.53]	23.67 [21.39, 25.80]	0.101	24.46 [22.56, 26.39]	23.91 [21.89, 25.52]	0.292
NYHA grade (%)			<0.001			0.594
I	39 (16.53)	7 (7.29)		6 (10.34)	7 (12.07)	
II	123 (52.12)	55 (57.29)		36 (62.07)	30 (51.72)	
III	73 (30.93)	27 (28.12)		15 (25.86)	18 (31.03)	
IV	1 (0.42)	7 (7.29)		1 (1.72)	3 (5.17)	
Hypertension (%)	152 (64.41)	52 (54.17)	0.107	32 (55.17)	36 (62.07)	0.572
Diabetes mellitus (%)	91 (38.56)	14 (14.58)	<0.001	12 (20.69)	13 (22.41)	1
CAD (%)	171 (72.46)	24 (25.00)	<0.001	17 (29.31)	21 (36.21)	0.553
AF (%)	19 (8.05)	20 (20.83)	0.002	8 (13.79)	8 (13.79)	1
Emergency surgery (%)	3 (1.27)	3 (3.12)	0.487	0 (0.00)	1 (1.72)	1
Type of surgery (%)			0.238			0.307
CABG (%)	130 (55.08)	57 (59.38)		18 (31.03)	14 (24.14)	
Cardiac valve surgery (%)	84 (35.59)	26 (27.08)		33 (56.90)	31 (53.45)	
Others (%)	22 (9.32)	13 (13.54)		7 (12.07)	13 (22.41)	
LVEF	61.00 [50.00, 66.00]	60.00 [51.75, 65.25]	0.75	60.00 [50.25, 66.75]	60.00 [56.00, 66.00]	0.943
Leukocyte (10^9^/L)	6.44 [5.03, 7.81]	5.76 [4.97, 7.28]	0.138	6.79 [5.20, 7.90]	5.76 [5.03, 7.22]	0.103
HCT	38.70 [35.68, 42.00]	39.80 [36.80, 43.23]	0.044	40.05 [36.47, 42.85]	39.90 [37.10, 43.58]	0.485
Platelets (10^9^/L)	200.50 [164.75, 243.25]	197.50 [169.50, 244.75]	0.694	215.50 [167.75, 260.50]	197.00[173.50, 241.25]	0.6
Neutrophils (10^9^/L)	3.70 [2.89, 5.03]	3.59 [2.73, 4.53]	0.422	3.90 [3.07, 5.72]	3.54 [2.71, 4.52]	0.323
Lymphocytes (10^9^/L)	1.68 [1.31, 2.08]	1.65 [1.28, 2.00]	0.779	1.74 [1.32, 2.24]	1.69 [1.31, 2.09]	0.776
PIV	231.95 [139.85, 409.42]	219.85 [117.50, 406.20]	0.747	255.40 [150.02, 481.98]	205.00[114.10, 410.60]	0.341
NLR	2.20 [1.60, 3.30]	2.30 [1.60, 3.10]	0.773	2.30 [1.63, 3.58]	2.25 [1.60, 3.15]	0.67
PLR	119.60 [93.30, 156.95]	125.75 [92.20, 159.92]	0.596	114.35 [94.03, 170.93]	126.70 [90.22, 158.27]	0.847
SII	453.00 [292.98, 655.05]	420.30 [278.20, 684.05]	0.854	464.75 [294.32, 785.32]	398.55[301.68, 672.55]	0.556
CRP (mg/L)	1.73 [1.60, 5.54]	2.21 [1.60, 6.84]	0.519	1.60 [1.60, 4.63]	1.60 [1.60, 4.80]	0.968
Albumin (g/L)	39.80 [37.20, 42.52]	39.90 [37.18, 42.32]	0.724	40.15 [37.60, 43.95]	40.40 [37.55, 42.95]	0.609
Creatinine (μmoI/L)	80.70 [67.00, 96.25]	85.00 [68.75, 102.70]	0.279	76.00 [65.88, 93.50]	83.65 [65.65, 92.60]	0.709
TnT (μg/L)	0.01 [0.01, 0.03]	0.02 [0.01, 0.02]	0.851	0.01 [0.01, 0.03]	0.02 [0.01, 0.02]	0.695
BNP (ng/ml)	422.10 [110.60, 1,243.75]	340.35 [104.25, 970.82]	0.415	422.10 [172.80, 1,162.25]	217.10 [97.12, 978.28]	0.284

CPB: cardiopulmonary bypass; BMI, body mass index; NYHA, New York Heart Association; CAD, coronary artery disease; AF, atrial fibrillation; CABG, coronary bypass surgery; LVEF, left ventricular ejection fraction; HCT, hematocrit; PIV, pan-immune inflammatory value; NLR, neutrophil/lymphocyte ratio; PLR, platelet/lymphocyte ratio; SII, systemic inflammation index; CRP, C-reactive protein; TnT, Troponin T; BNP, brain natriuretic peptide.

Data are presented as median [P25, P75] or *n* (%).

### Comparison of intraoperative data and postoperative clinical outcomes between the two groups

Intraoperative data and postoperative clinical outcomes for the two groups included in the final analysis were compared before and after PSM. Compared with the non-CPB group, the CPB group had a higher proportion of intraoperative transfusion of blood products (63.79% vs. 6.90%, *P* < 0.001), specifically for red blood cells (58.62% vs. 3.45%, *P* < 0.001) and plasma (41.38% vs. 1.72%, *P* < 0.001). In addition, the CPB group had a higher volume of drainage fluid within 24 h [380 (200–550) ml vs. 200 (24–330) ml, *P* = 0.002] and required longer durations of mechanical ventilation [14.3 (6.6–21.3) h vs. 5.75 (4.08–10.1) h, *P* < 0.001] and ICU stay [48.78 (44.92–89.38) h vs. 27.16 (21.67–46.25) h, *P* < 0.001]. However, there was no significant difference in mortality between the two groups (Table 2).

**Table 2:** Comparison of intraoperative data and postoperative clinical outcomes between two groups.

Variable	Overall population		*P*-value	Propensity score matched population		*P*-value
	Non-CPB Group (*n* = 236)	CPB group (*n* = 96)		Non-CPB group (*n* = 58)	CPB Group (*n* = 58)	
Intraoperative total transfusion, *n* (%)	29 (12.29)	61 (63.54)	<0.001	4 (6.90)	37 (63.79)	<0.001
RBC transfusion, *n* (%)	18 (7.63)	58 (60.42)	<0.001	2 (3.45)	34 (58.62)	<0.001
Plasma transfusion, *n* (%)	5 (2.12)	35 (36.46)	<0.001	1 (1.72)	24 (41.38)	<0.001
Platelet transfusion, *n* (%)	0 (0.00)	3 (3.12)	0.037	0 (0.00)	1 (1.72)	1.000
IABP assist, *n* (%)	0 (0.00)	2 (2.08)	0.149	0	0	N/A
AKI, *n* (%)	51 (21.61)	20 (20.83)	1.000	11 (18.91)	14 (24.14)	0.652
CRRT, *n* (%)	4 (1.69)	3 (3.12)	0.688	2 (3.45)	2 (3.45)	1.000
Postoperative infection, *n* (%)	17 (7.20)	20 (20.83)	0.001	3 (5.17)	10 (17.24)	0.077
Drainage fluid volume within 24 h (ml)	330 [166, 472]	355 [177, 555]	0.355	200 [24, 330]	380 [200, 550]	0.002
Lowest P/F within 48 h (mmHg)	213 [172, 251]	206[163, 229]	0.386	204 [156, 238]	208 [168, 246]	0.665
Mechanical ventilation duration (h)	315 [230, 551]	765[381, 1,350]	<0.001	5.75 [4.08, 10.1]	14.3 [6.6, 21.3]	<0.001
ICU stay duration (h)	32.99 [21.85, 46.33]	47.00 [43.38, 91.18]	<0.001	27.16 [21.67, 46.25]	48.78 [44.92, 89.38]	<0.001
Death *n* (%)	3 (1.27)	6 (6.25)	0.031	0 (0)	2 (3.45)	0.476

CPB, cardiopulmonary bypass; RBC, red blood cell; IABP, intra-aortic balloon pump; AKI, acute kidney injury; CRRT, continuous renal replacement therapy; P/F, arterial partial oxygen pressure/oxygen absorption concentration; ICU, intensive care unit.

Data are presented as median [P25, P75] or *n* (%).

### Comparison of postoperative clinical laboratory findings between the two groups

In terms of inflammation biomarkers, on the day after surgery, NLR was significantly higher in the CPB group [14.00 (9.93–23.08) vs. 11.55 (7.38–17.38), *P* = 0.043], whereas there were no significant differences in other indicators. On the first postoperative day, the non-CPB group exhibited higher values for several indicators: including PIV [2,220.45 (1,199.15–2,970.43) vs. 1,563.00 (738.50–2,944.78), *P* = 0.039], PLR [258.10 (178.75–363.20) vs. 202.15 (134.07–271.58), *P* = 0.003], and SII [2,434.90 (1,611.05–3,631.80) vs. 1,793.40 (1,200.65–2,829.33), *P* = 0.01]. Nevertheless, no statistical difference in NLR was found between the two groups on that day (Table 3).

**Table 3:** Comparison of postoperative clinical laboratory findings between two groups.

Variable	Overall population		*P*-value	Propensity score matched population		*P*-value
	Non-CPB group (*n* = 236)	CPB group (*n* = 96)		Non-CPB group (*n* = 58)	CPB group (*n* = 58)	
Postoperative laboratory findings (day 0)						
Leukocytes (10^9^/L)	9.55 [6.94, 12.13]	11.75 [8.40, 15.18]	<0.001	10.09 [6.07, 12.63]	11.16 [7.51, 13.82]	0.200
HCT (%)	31.55 [28.50, 34.60]	30.55 [28.37, 33.00]	0.026	32.65 [28.40, 34,82]	30.40 [27.55, 32.70]	0.030
Platelets (10^9^/L)	164.00 [135.00, 201.00]	116.50 [97.75, 157.25]	<0.001	157.00 [132.25, 195.25]	116.50 [95.75, 158.75]	<0.001
Neutrophils (10^9^/L)	8.28 [5.82, 10.82]	10.57 [7.38, 13.41]	<0.001	8.68 [4.93, 11.40]	9.88 [6.78, 11.95]	0.165
Lymphocytes (10^9^/L)	0.69 [0.51, 0.93]	0.72 [0.43, 1.42]	0.563	0.69 [0.51, 1.02]	0.66 [0.37, 0.95]	0.279
PCT (ng/ml)	0.05 [0.03, 0.10]	0.06 [0.04, 0.11]	0.559	0.05 [0.03, 0.10]	0.06 [0.04, 0.12]	0.358
CRP (mg/L)	1.60 [1.60, 4.30]	2.06 [1.60, 7.29]	0.017	1.60 [1.60, 4.29]	1.72 [1.60, 4.46]	0.602
PIV	554.90 [296.88, 1,197.23]	790.80 [380.25, 1, 311.88]	0.114	560.20 [326.82, 1,633.50]	858.35 [441.52, 1,299.42]	0.397
NLR	11.40 [7.47, 17.52]	13.20 [8.40, 21.48]	0.08	11.55 [7.38, 17.38]	14.00 [9.93, 23.08]	0.043
PLR	235.10 [167.52, 336.18]	177.40 [92.17, 277.88]	<0.001	242.35 [145.25, 323.42]	196.60 [103.65, 324.35]	0.224
SII	1,796.35 [1,074.80, 2,867.17]	1,645.10 [1,010.15, 2,609.55]	0.135	1,867.05 [1,023.80, 2,719.75]	1,938.55 [1,182.43, 2,682.98]	0.963
Postoperative laboratory findings (day 1)						
Leukocytes (10^9^/L)	11.92 [9.80, 14.41]	11.30 [9.11, 13.99]	0.143	11.39 [9.78, 14.49]	11.25 [8.91, 13.73]	0.481
HCT (%)	32.80 [29.30, 35.32]	30.20 [27.10, 33.42]	<0.001	33.40 [29.45, 35.62]	30.30 [26.95, 34.00]	0.009
Platelets (10^9^/L)	174.00 [135.00, 210.25]	116.50 [97.75, 157.25]	0.279	157.50 [135.00, 190.50]	118.50 [92.25, 153.50]	<0.001
Neutrophils (10^9^/L)	10.18 [8.37, 12.50]	9.93 [7.86, 11.53]	0.174	9.74 [7.84, 12.67]	9.88 [7.60, 11.67]	0.633
Lymphocytes (10^9^/L)	0.71 [0.50, 0.97]	0.62 [0.43, 0.81]	0.029	0.62 [0.42, 0.82]	0.64 [0.44, 0.81]	0.825
PCT (ng/ml)	0.75 [0.29, 2.14]	0.59 [0.28, 2.45]	0.676	0.58 [0.33, 1.36]	0.47 [0.24, 2.67]	0.766
CRP (mg/L)	57.83 [30.49, 83.80]	55.10 [32.48, 81.74]	0.667	49.04 [30.49, 85.89]	40.99 [29.52, 63.28]	0.401
PIV	2,230.25 [1,309.98, 3,472.70]	1,605.45 [870.50, 2,714.90]	0.001	2,220.45 [1,199.15, 2,970.43]	1,563.00 [738.50, 2,944.78]	0.039
NLR	14.30 [10.47, 21.22]	15.20 [11.47, 22.52]	0.247	15.75 [10.93, 25.73]	15.05 [11.82, 19.90]	0.770
PLR	243.55 [174.45, 346.62]	202.15 [134.23, 284.10]	0.001	258.10 [178.75, 363.20]	202.15 [134.07, 271.58]	0.003
SII	2,453.90 [1,615.92, 3,666.35]	1,854.65 [1,236.30, 2,888.10]	<0.001	2,434.90 [1,611.05, 3,631.80]	1,793.40 [1,200.65, 2,829.33]	0.010

CPB, cardiopulmonary bypass; HCT, hematocrit; PCT, procalcitonin; CRP, C-reactive protein; PIV, pan-immune inflammatory value; NLR, neutrophil/lymphocyte ratio; PLR, platelet/lymphocyte ratio; SII, systemic inflammation index.

Data are presented as median [P25, P75] or *n* (%).

### Comparison of perioperative inflammatory biomarkers in the CPB group

Perioperative inflammation biomarkers in the CPB group were compared. All novel inflammatory biomarkers were significantly elevated compared to presurgery levels, peaking on the first day after surgery and then entering a downward trend (Table 4).

**Table 4:** Comparison of perioperative inflammation biomarkers in the CPB group.

Variable	Preoperative (*n* = 96)	Postoperative day 0 (*n* = 96)	Postoperative day 1 (*n* = 96)	Postoperative day 2 (*n* = 96)	*P*-value
Leukocytes (10^9^/L)	5.76 [4.97, 7.28]	11.75 [8.40, 15.18]	11.30 [9.11, 13.99]	13.23 [10.19, 15.65]	<0.001
Neutrophils (10^9^/L)	3.59 [2.73, 4.53]	10.57 [7.38, 13.41]	9.93 [7.86, 11.53]	11.18 [8.55, 13.06]	<0.001
Lymphocytes (10^9^/L)	1.65 [1.28, 2.00]	0.72 [0.43, 1.42]	0.62 [0.43, 0.81]	0.87 [0.66, 1.12]	<0.001
Platelets (10^9^/L)	197.50 [169.50, 244.75]	116.50 [97.75, 157.25]	118.50 [94.75, 150.50]	114.00 [82.00, 136.25]	<0.001
CRP (mg/L)	2.21 [1.60, 6.84]	2.06 [1.60, 7.29]	55.10 [32.48, 81.74]	133.22 [98.32, 164.52]	<0.001
PIV	219.85 [117.50, 406.20]	790.80 [380.25, 1,311.88]	1,605.45 [870.50, 2,714.90]	1,328 [810.95, 2,183.85]	<0.001
NLR	2.30 [1.60, 3.10]	13.20 [8.40, 21.48]	15.20 [11.47,22.52]	12.40 [9.38, 15.87]	<0.001
PLR	125.75 [92.20, 159.92]	177.40 [92.17, 277.88]	202.15 [134.23, 284.10]	129.15 [87.90, 178.57]	<0.001
SII	420.30 [278.20, 684.05]	1,645.10 [1,010.15, 2,609.55]	1,854.65 [1,236.30, 2,888.10]	1,348.45 [905.40, 1,938.62]	<0.001

CRP, C-reactive protein; PIV, pan-immune inflammatory value; NLR, neutrophil/lymphocyte ratio; PLR, platelet/lymphocyte ratio; SII, systemic inflammation index.

Data are presented as median [P25, P75].

### Predictive value of inflammatory markers for postoperative infection and AKI

**Figure 2:**
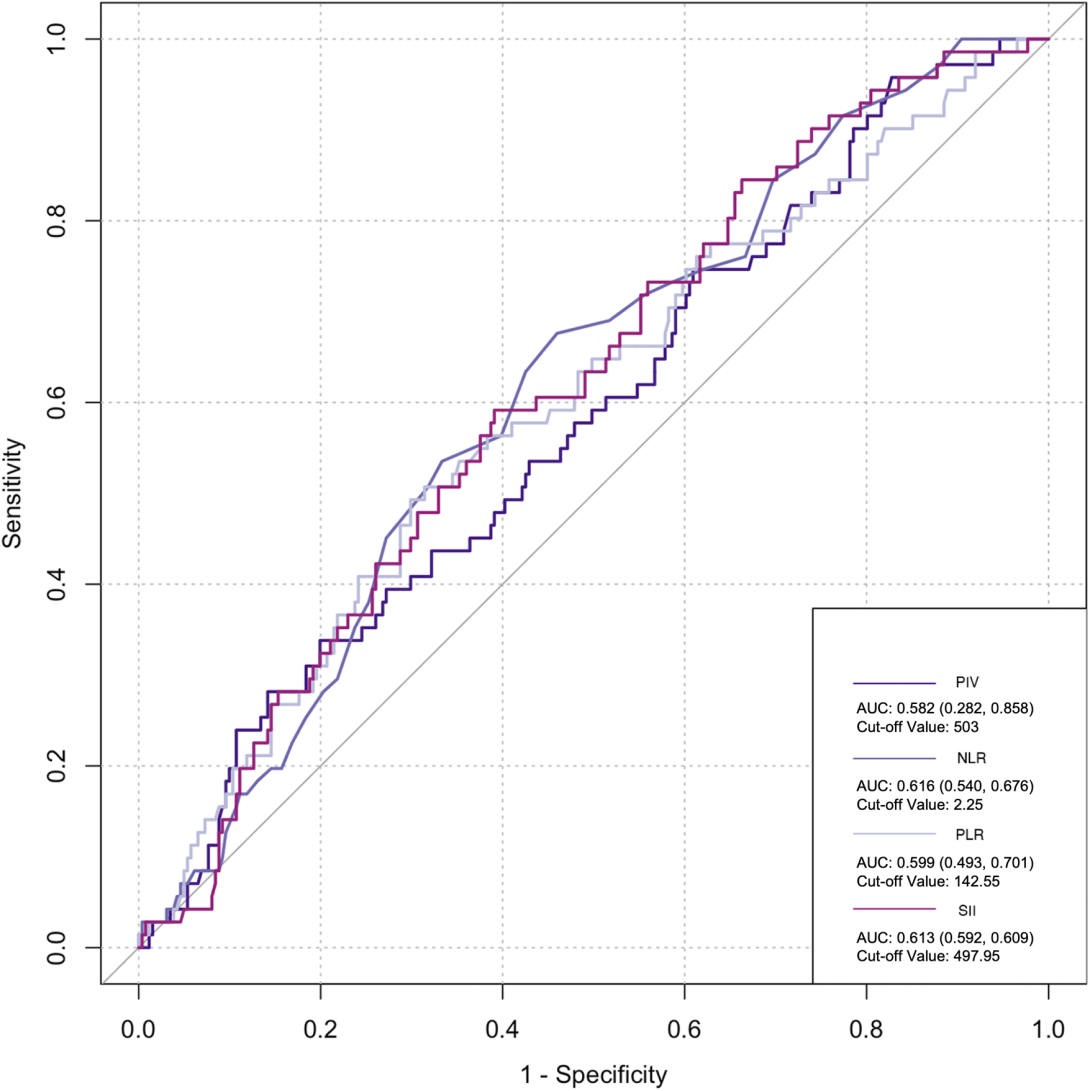
Preoperative biomarkers’ predictive value for AKI.

**Figure 3:**
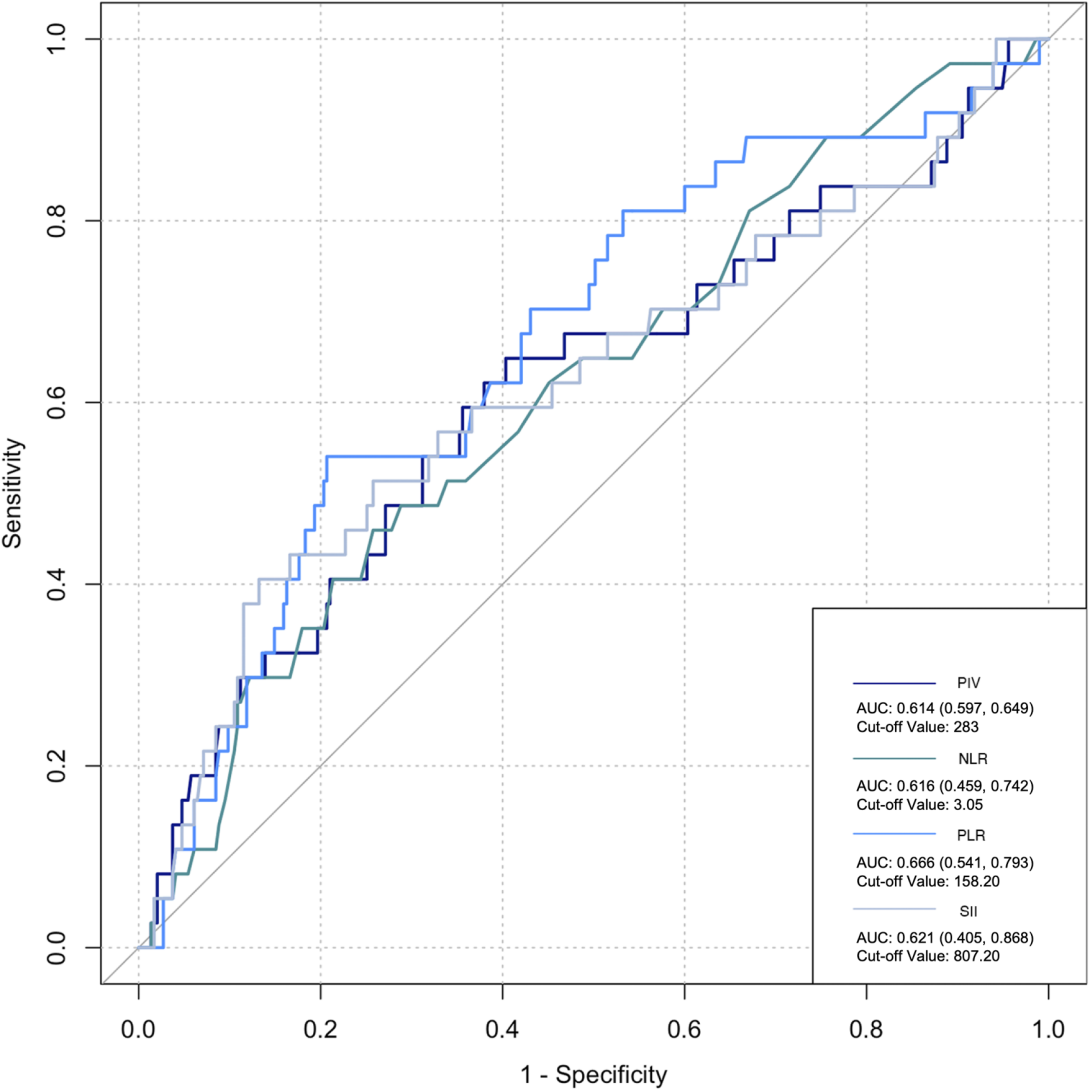
Preoperative biomarkers’ predictive value for infection.

**Figure 4:**
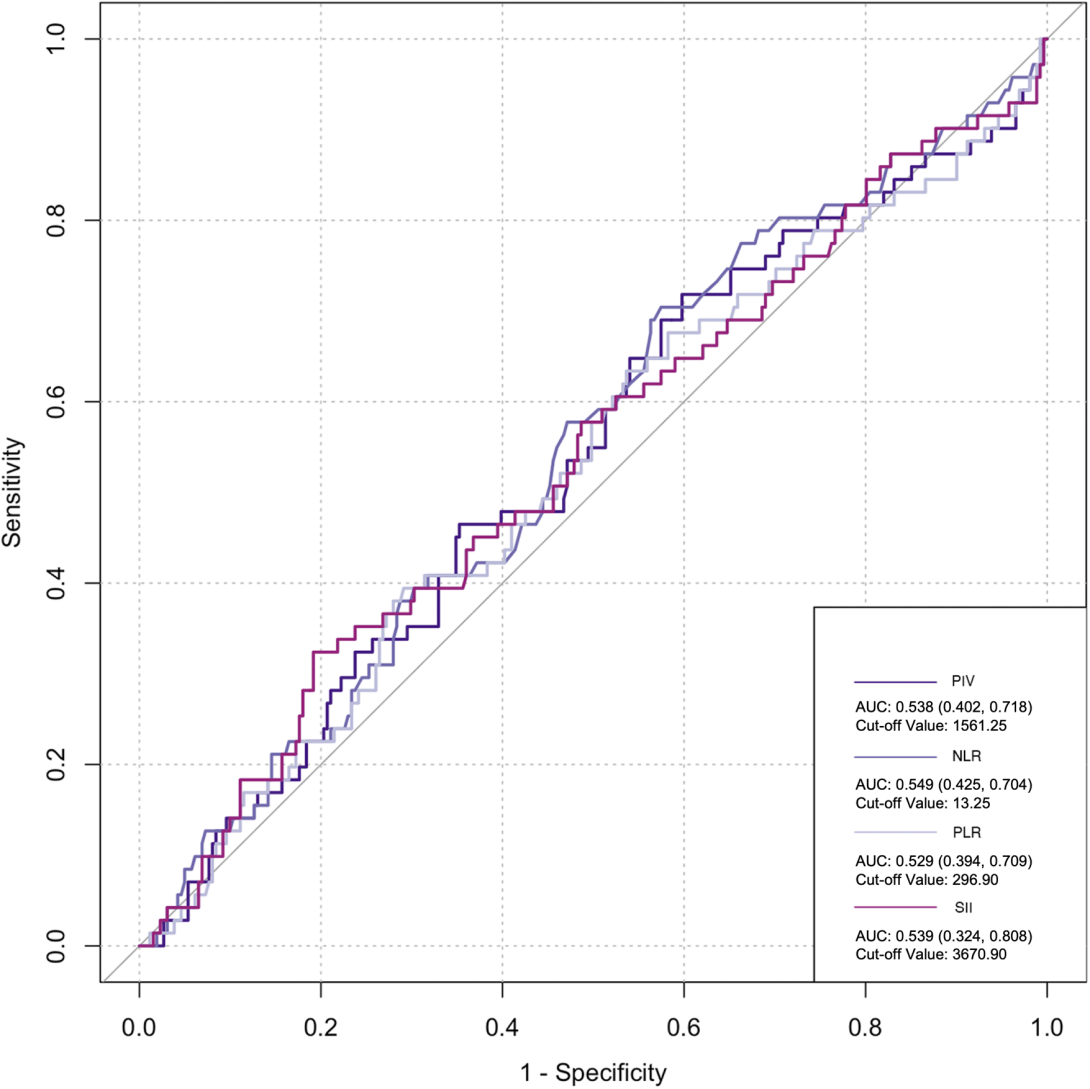
Postoperative biomarkers’ predictive value for AKI.

**Figure 5:**
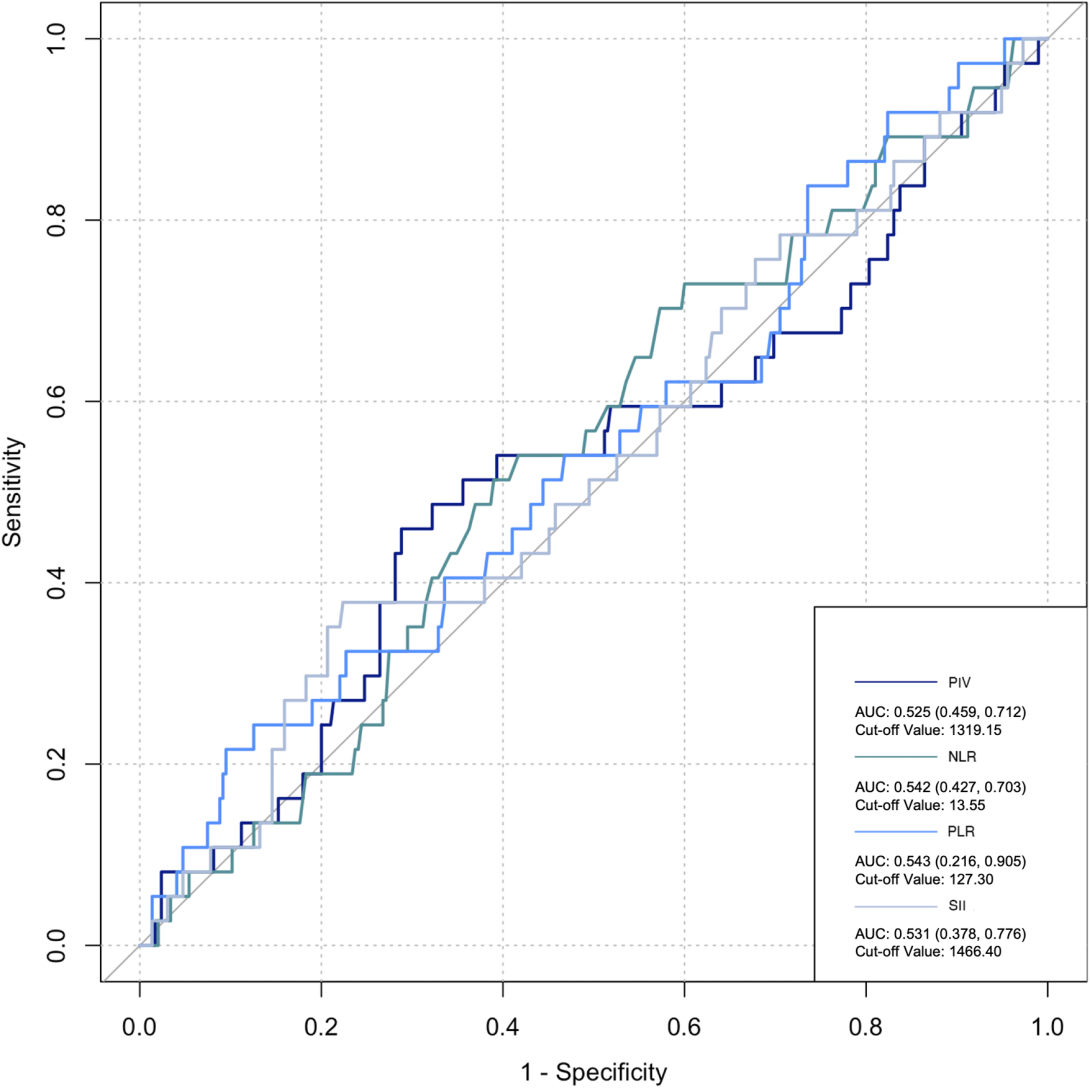
Postoperative biomarkers’ predictive value for infection. PIV, pan-immune inflammatory value; NLR, neutrophil/lymphocyte ratio; PLR, platelet/lymphocyte ratio; SII, systemic inflammation index.

The ROC curve was employed to assess the predictive ability of postoperative infection and AKI. Among the inflammatory biomarkers analyzed, namely, PIV, PLR, and SII, their predictive values were not notably high. Specifically, only preoperative NLR demonstrated moderate predictive power, with an AUC of 0.616 [95% confidence interval (CI): 0.459–0.742] for postoperative infection, using a cutoff value of 3.05. Similarly, for postoperative AKI, preoperative NLR also yielded an AUC of 0.616 (95% CI: 0.54–0.676), with a cutoff value of 2.25 (Figures 2–5).

## Discussion

Currently, the majority of open cardiovascular surgeries and extensive vascular procedures still involve CPB. Most patients exhibit a systemic inflammatory response after CPB, characterized by elevated levels of circulating inflammatory cytokines and the activation of inflammatory cells (5). Clinical investigations have revealed a profound correlation between the intensity of this inflammatory response and unfavorable patient outcomes (6). Our investigation further indicates that individuals undergoing CPB surgery are at a higher risk of requiring perioperative blood transfusions and experiencing increased postoperative drainage within the first 24 h. In addition, these patients often require prolonged mechanical ventilation support and extended ICU stays due to the physiological impact of CPB. However, our study did not detect any significant increase in the likelihood of postoperative complications, such as AKI or infection, among patients who underwent CPB surgery. Moreover, there was no significant difference in the in-hospital mortality rate between the CPB and non-CPB groups.

Formerly, within the cardiovascular field, cellular inflammatory markers, including interleukin-6 (IL-6), tumor necrosis factor (TNF), C-reactive protein (CRP), and procalcitonin (PCT), have been extensively researched. Notably, an elevated IL-6 level above 421 pg/ml has been associated with a substantial increase in the risk of postoperative mortality among patients (OR = 12.6, 95% CI: 2.96–53.55) (7). In addition, IL-6 is a reliable predictor of postoperative delirium (8). IL-6 levels surge immediately following the commencement of CPB (9, 10), peaking at the end of surgery and gradually returning to baseline levels by the third postoperative day (11). On the other hand, CRP levels peak 48 h postoperatively, followed by a decline after 72 h, with a maximum value of 58.82 ± 42.23 mg/L, representing a 3–10-fold increase from baseline (12). Conventionally, CRP is considered more sensitive for the early diagnosis of inflammation, and a higher concentration often indicates a poorer prognosis for patients (13). However, some studies have contradicted this notion, revealing no significant correlation between elevated CRP levels and the occurrence of postoperative inflammation or clinical outcomes (14).

PCT, primarily utilized for the early diagnosis of infection, attains its peak value within 24 h postoperatively (15), averaging at 0.77 ± 0.49 ng/ml, approximately two to four times higher than the baseline level (12); however, its concentration levels are also positively associated with organ dysfunction. Studies have shown that patients with PCT >2.5 ng/L have a 4.5-fold increase in mortality at 28 days after surgery (16). When PCT concentrations exceed 0.7 ng/ml, postoperative organ failure can be predicted (with a sensitivity of 85% and specificity of 58%), while when PCT concentrations exceed 7.7 ng/ml, both sensitivity and specificity reach 100% (17). Serum PCT concentrations in patients with multiple organ dysfunction can reach 20 ng/ml (18). These inflammatory markers, although informative, require careful interpretation in the context of individual patient characteristics and surgical procedures to ensure accurate prognostication and tailored treatment strategies, especially in cardiovascular surgery.

In recent years, numerous research studies have focused on developing novel inflammatory markers by integrating multiple biomarkers, including the PLR, NLR, SII, and PIV. These biomarkers have demonstrated unique roles in various studies. Many factors can affect the occurrence of AKI after cardiac surgery, such as preoperative neopterin levels, EuroSCORE II, and clamp time, all of which have been identified as independent predictors of postoperative AKI (19). Previous studies have highlighted the NLR on the first postoperative day as a robust and independent predictor of early AKI in patients undergoing isolated off-pump coronary artery bypass (OPCAB) (20). Meanwhile, PIV has garnered significant attention in the context of ST-elevation myocardial infarction (STEMI). Recently, relevant studies have expanded the application of PIV to patients with acute heart failure and STEMI, establishing its close association with patient prognosis in cardiology (21, 22). However, there is relatively little research on these indicators in the cardiovascular surgery field. Our study showed that novel inflammatory biomarkers consistently increase after cardiovascular surgeries involving CPB, peaking on the first day after surgery. However, compared to non-CPB cardiovascular surgery, our analysis of PLR, SII, and PIV revealed a paradoxical finding: a seemingly attenuated postoperative inflammatory response in the CPB group. This observation is clearly in contrast with reality and is inconsistent with our impression. A careful analysis suggests that this discrepancy may be closely linked to the destruction of platelets during CPB. Current understanding suggests that platelet destruction, adhesion, and aggregation during CPB are primary factors contributing to reduced platelet counts after surgery (23). Prolonged CPB time further increases the risk of postoperative thrombocytopenia (24). Given these considerations, NLR, without the confounding influence of platelets, has emerged as a more informative metric for assessing perioperative inflammatory responses.

Previous studies have revealed that inflammatory cells contribute significantly to organ damage following CPB, primarily through two distinct mechanisms. Initially, monocytes adhere to vascular endothelial cells by upregulating CD11b expression and subsequently migrate from the blood vessels into tissues. Upon reaching the tissues, these monocytes upregulate the production of various inflammatory cytokines, including IL-6, IL-8, and TNF-α, creating a localized high-concentration zone (25). Notably, the concentration of these cytokines differs significantly from that observed intravascularly. A portion of these locally produced soluble factors enters the circulation, activating other immune effector cells, such as neutrophils. These activated neutrophils, guided by chemotactic signals from high-concentration cytokines in the tissue, migrate to the inflamed tissues through upregulated surface adhesion molecules like Mac-1 (CD11b/CD18). Upon arrival, they release oxygen free radicals, myeloperoxidase, elastase, and other agents, causing damage to surrounding tissues (26). Collectively, these observations suggest that monocytes and neutrophils are the primary immune effector cells mediating systemic inflammatory response (SIR) induced by CPB. Notably, the inclusion of more indices in research introduces more interfering factors, particularly considering the damage to platelets and blood cells caused by CPB, thus leading to potential data deviations. However, NLR solely comprises the ratio of neutrophils to lymphocytes. Neutrophils play an important role in inflammation, while lymphocyte count reflects physiological stress and is negatively correlated with inflammation (27). Dynamic changes in NLR are attributed to systemic inflammation. High NLR significantly increases the risk of mortality, postoperative re-intubation, and atrial fibrillation after cardiovascular surgery (28, 29). A meta-analysis estimated an AUC of 0.65 for NLR in predicting AKI following cardiovascular surgery (30). Lafçi et al. emphasized the utility of NLR as a simple and effective marker for predicting outcomes in a high-risk cardiovascular cohort (31), which is consistent with our research. In the present study, ROC curve analysis yielded a similar prediction for postoperative AKI and infection with NLR (AUC: 0.616), supporting NLR as a potentially reliable prognostic indicator.

## Limitations

Our study, despite its valuable contributions, has several limitations that need to be addressed. First, the clinical data collected for the study may not be comprehensive enough to provide a complete picture of the subject matter. This could be due to various reasons such as limited access to patient records, incomplete reporting of symptoms, or the absence of certain critical information. Second, the results obtained from this study may not be generalizable to other populations, given the specific characteristics of the sample population and the conditions under which the study was conducted. To overcome these limitations and further validate the findings of this study, larger, randomized controlled trials are urgently needed. Such trials would involve a more diverse and representative sample population, allowing for more robust and generalizable results. In addition, the use of randomized allocation of participants to different treatment groups would help minimize potential biases and confounding factors, ensuring that the observed effects are truly attributable to the intervention being studied. By conducting such rigorous trials, we can gain a deeper understanding of the subject matter and make more informed decisions about the effectiveness of different treatment options.

## Conclusions

In conclusion, patients undergoing cardiovascular surgery with CPB exhibit a poorer prognosis compared to those without CPB. Novel inflammatory biomarkers, including PLR, PIV, and SII, may offer valuable insights into the degree of postoperative inflammation, with NLR emerging as a potentially reliable prognostic indicator. Certainly, these findings necessitate further rigorous research and validation.

## Data Availability

The original contributions presented in the study are included in the article/Supplementary Material, further inquiries can be directed to the corresponding authors.
